# Diffusion Tensor Tractography versus Volumetric Imaging in the Diagnosis of Behavioral Variant Frontotemporal Dementia

**DOI:** 10.1371/journal.pone.0066932

**Published:** 2013-07-18

**Authors:** Alexander Frizell Santillo, Johanna Mårtensson, Olof Lindberg, Markus Nilsson, Amir Manzouri, Maria Landqvist Waldö, Danielle van Westen, Lars-Olof Wahlund, Jimmy Lätt, Christer Nilsson

**Affiliations:** 1 Clinical Memory Research Unit, Department of Clinical Medicine, Lund University, Lund, Sweden; 2 Geriatric Psychiatry, Department of Clinical Medicine, Lund University, Lund, Sweden; 3 Department of Radiology, Oncology and Radiation Sciences, Uppsala University, Uppsala, Sweden; 4 Division of Clinical Geriatrics, Karolinska Institute, Stockholm, Sweden; 5 Lund University Bioimaging Center LBIC, Lund University, Lund, Sweden; 6 Center for Medical Imaging and Physiology, Skåne University Hospital, Lund, Sweden; Beijing Normal University, China

## Abstract

MRI diffusion tensor imaging (DTI) studies of white matter integrity in behavioral variant frontotemporal dementia have consistently shown involvement of frontal and temporal white matter, corresponding to regional loss of cortical volume. Volumetric imaging has a suboptimal sensitivity as a diagnostic tool and thus we wanted to explore if DTI is a better method to discriminate patients and controls than volumetric imaging. We examined the anterior cingulum bundle in 14 patients with behavioral variant frontotemporal dementia and 22 healthy controls using deterministic manual diffusion tensor tractography, and compared DTI parameters with two measures of cortical atrophy, VBM and cortical thickness, of the anterior cingulate cortex (ACC). Statistically significant changes between patients and controls were detected in all DTI parameters, with large effect sizes. ROC-AUC was for the best DTI parameters: 0.92 (fractional anisotropy) to 0.97 (radial diffusivity), 0.82 for the best cortical parameter, VBM of the ACC. Results from the AUC were confirmed with binary logistic regression analysis including demographic variables, but only for fractional anisotropy and mean diffusivity. Ability to classify patient/nonpatient status was significantly better for mean diffusivity vs. VBM (p=0.031), and borderline significant for fractional anisotropy vs. VBM (p=0.062). The results indicate that DTI could offer advantages in comparison with the assessment of cortical volume in differentiating patients with behavioral variant frontotemporal dementia and controls.

## Introduction

Behavioral variant frontotemporal dementia (bvFTD) is a neurodegenerative condition affecting the frontal and temporal lobes. Characteristic symptoms of bvFTD are behavioral disinhibition, concurrent with apathy, repetitive behavior and hyperorality [1,2]. Currently a diagnosis of probable bvFTD relies on identifying a number of these symptoms and to attribute these to the frontotemporal neurodegenerative process, either by structural or functional neuroimaging [2]. Morphological MRI has a suboptimal sensitivity in the initial assessment (up to 65%) [3,4], while functional imaging is more sensitive [3] and thus recommended in diagnostic flowcharts if morphological MRI is normal [1]. Clearly, a more sensitive MRI modality could be of use here [5]. Traditional diagnostic MRI is based on the radiologist grading, often semi-quantitatively, cortical atrophy on visual inspection of morphological images. DTI is an MRI modality that allows for visualization of white matter integrity; with DTI it has been shown that diffusion parameters are changed in frontal and temporal white matter in patients with bvFTD compared with controls and patients with Alzheimer’s disease [6]. These changes have most consistently been replicated in the anterior part of the corpus callosum, the anterior part of the cingulum bundle, and in the uncinate fasciculus [7–11]. To a lesser extent, changes have also been seen in the anterior parts of the inferior longitudinal fasciculus and the superior longitudinal *Fasciculus* [7]. Changes in white matter diffusion seem to correlate with atrophy of corresponding gray matter regions [7,8,10,12,13]. This is well in accordance with findings from neuropathology, which show that white matter degeneration accompanies gray matter degeneration in FTD [14]. On MRI (T2 or FLAIR) this pathology is seen as white matter hyperintensities (WMH) [15,16]. To examine the possible diagnostic advantage of DTI versus the assessment of cortical volume, which is the purpose of this study, it is necessary to examine these parameters in individual patients. This has previously been performed in one study of bvFTD, using a whole brain voxel-wise comparison approach, showing that a gray matter loss in the frontomedian cortex was less accurate to predict diagnosis than diffusion parameters in the corresponding region and in the cingulum bundle, as determined by a tract of interest approach [17].

In this study we perform an anatomically detailed analysis of a specific region, the cingulum bundle, using manual deterministic diffusion tensor tractography, and we compare this with atrophy of the anterior cingulate cortex (ACC), assessed by two different methods. We chose to investigate the ACC and the cingulum bundle since the ACC is one of the regions first affected by atrophy in bvFTD and is thus of particular interest for early diagnosis [18]. It is also one of the cortical regions most consistently affected in bvFTD [19–21] and is affected in all bvFTD genetic mutation types [22]. Due to the anatomical correspondence between the ACC and the cingulum bundle [23,24] these structures are particularly suitable for the purpose of making a comparison between volume change and DTI parameters. The cingulum bundle does contain traversing fibers that connect to cortical areas other than the ACC, but most fibers are efferents or afferents destined for parts of the cingulate cortex [23,24]. This relationship between the cortex and white matter is not that straightforward in the case of other areas commonly and/or early affected in bvFTD, such as the anterior temporal cortex, the orbitofrontal cortex, the insula, or other part of the median frontal cortex other than the ACC. The cingulate cortex and the cingulum bundle are well described anatomically both from post-mortem-tracing methodology and DTI [23–25]: the cingulum bundle conveys fibers to/from the cingulate cortex destined for orbitofrontal, dorsolateral frontal, anterior insular cortices, posteriorly to the parietal cortex and ventrally to the anterior temporal lobe. The cingulum bundle also has rich connections with the basal ganglia and thalamus, and a small portion of the cingulum bundle is commissural. The cingulum bundle contains a mix of long and short fibers (“U fibers”), which connect regions within the ACC and connect the ACC with adjacent cortical gyri [23,25]. These U fibers are of particular interest in the current context, since they are characteristically affected in FTD [14].

The purpose of this study was to examine the potential to discriminate patients with bvFTD from healthy controls, using measurements of gray and white matter of the ACC/cingulum bundle. White matter was assessed by extracting DTI parameters from diffusion tensor tractography of the cingulum bundle, whereas the cortical pathology was assessed using two different methods: one parcellation-based method (Freesurfer), and one VBM approach (VBM-FSL). Although VBM methods are most commonly used to compare group data, we used here a VBM approach to assess the cortical integrity of the ACC in individual cases, in order to compare the discriminatory potential of the different methods in separating patients and controls. Our results indicate that DTI could indeed offer advantages in the diagnosis of bvFTD.

## Methods

### Patients

All cases are from the Lund Prospective Frontotemporal Dementia Study, a longitudinal study of patients with a diagnosis or suspected diagnosis of any of the frontotemporal dementia spectrum disorders, although this study only covers bvFTD patients. The study inclusion criteria were: a clinical diagnosis or a suspected clinical diagnosis of bvFTD after standard clinical work up and that the patient should be capable of performing at least 2 of the following study procedures: lumbar puncture, MRI according to study protocol and neuropsychological examination. The clinical dementia rating scale [26], with the clinical dementia frontotemporal lobe degeneration addendum [27], and the frontal behavioral inventory [28] were administered, together with a standardized neurological examination. All data reported here are based on baseline data. Fourteen patients from the cohort had a diagnosis of probable (n= 10) or possible (n=4) bvFTD according to international criteria [2] and were included in the present study. The distinction between probable and possible in our cases depended on the results of the MRI, i.e. whether there was visual atrophy or not as determined by a neuroradiologist (DvW). WHM were rated by a neuroradiologist (DvW) according to the scales by Fazekas [29] and Wahlund [30] on FLAIR images. Data of bvFTD patients are presented in Table 1. None of the patients had CSF biomarker profiles indicative of Alzheimer’s disease. Screening for genetic mutations was not performed. One patient had a diagnosis of bvFTD with motor neuron disease. Healthy controls (n=22) were recruited from spouses of patients and underwent clinical interview and examination, including routine neuropsychological testing and MRI. Data for the healthy controls are presented in Table 1.

**Table 1:** Demographic data of healthy controls and patients with behavioral variant frontotemporal dementia (bvFTD).

	Healthy Controls	bvFTD
Number	22	14
Sex	10M 12F	7M 7F
Age	68.5 (55-82)	71.5 (38-78)
Education	11 (8-14)	8 (7-14)
MMSE	29.5 (29-30)	26 (12-30)
Duration	n/a	3 (1-12)
CDR-SB	n/a	7.3 (1-12.5)
FTLD-CDR	n/a	9.75 (2-16.5)
FBI	n/a	27.5 (11-37)

M: male, F: female. Age: age in years. Education: education in years. MMSE: Mini Mental Status Examination. Duration: years since symptom onset. CDR-SB: Clinical Dementia Rating Sum of Boxes. FTLD-CDR: Frontotemporal Lobar Degeneration modified Clinical Dementia Rating, Sum of Boxes. FBI: Frontal Behavioral inventory, total score, FBI 1-12: sum of FBI item 1 to 12, FBI 12-22: sum of FBI item 12 to 22. All values are median values, with range.

### Ethics statement

The study was approved by the Regional Ethical Review Board, Lund, Sweden (Number 617/2008). Patients and healthy controls were informed of the study content in both oral and written form. Informed consent was taken in written form. All subjects received a copy of the study information and their written informed consent.

### Data acquisition

MRI was performed using a Philips Achieva 3T scanner, equipped with an eight channel head coil. DTI data were acquired using a single-shot spin echo sequence with EPI, using 48 diffusion encoding directions, a diffusion-weighting factor (b) of 800 s/mm^2^, voxel size 2x2x2 mm, TR 7881 ms, TE 90 ms and an acquisition time of 6 min 49 s. Motion and eddy current correction of the data was performed using FLIRT [31]. Streamline tractography was performed with an FA threshold of 0.2, an angular threshold of 45 degrees and no length threshold. Data analysis was performed using the Diffusion Toolkit and TrackVis (version 0.5.0), which is available at http://www.trackvis.org. TrackVis was also used for positioning of ROIs and anatomical landmarks. The DTI protocol was followed by a morphological high resolution T1 3D sequence.

### Tractography and post-processing

**Figure 1:**
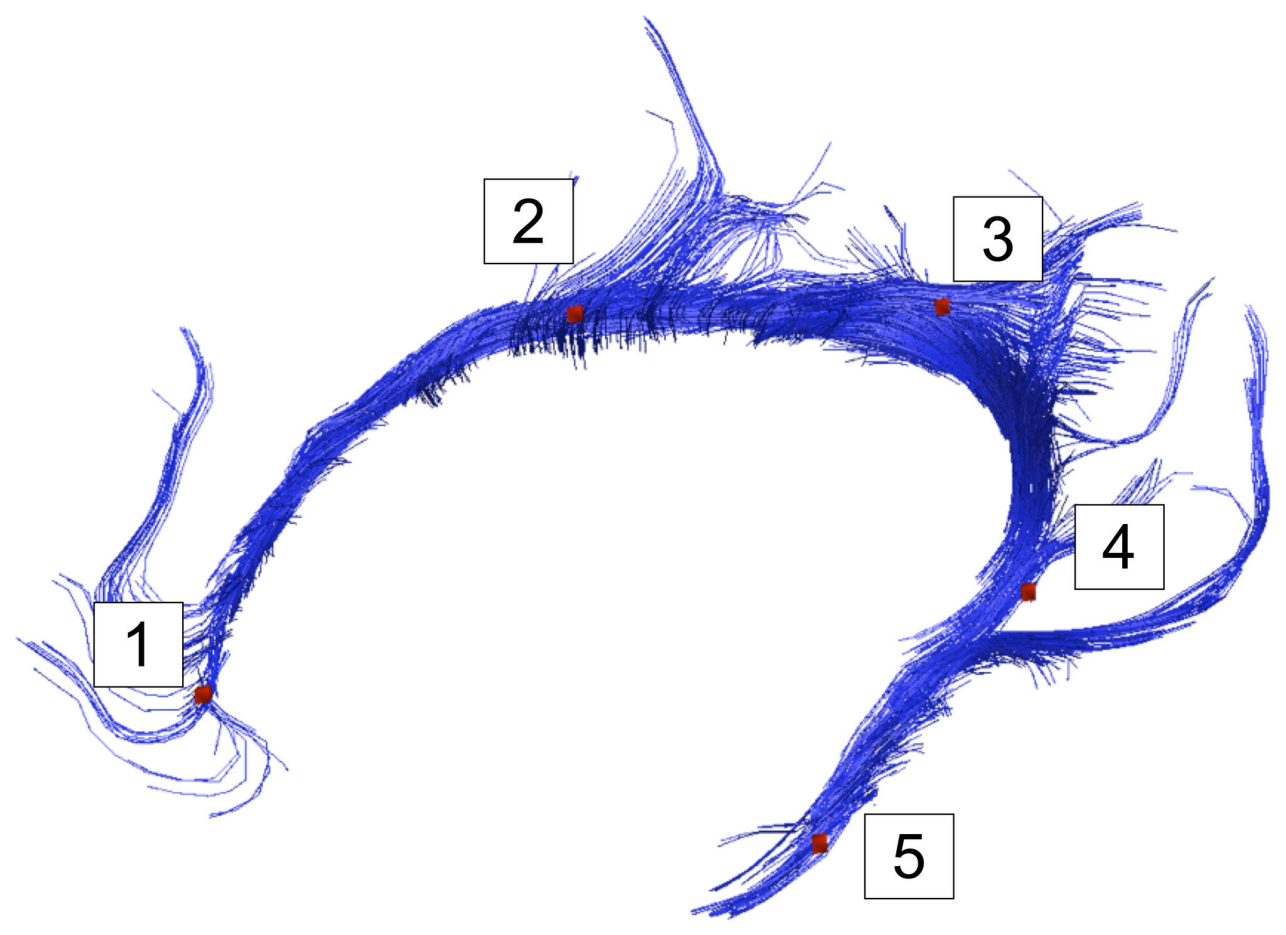
Tractography of the cingulum bundle. Graphical rendering of tractography of the left cingulum bundle (CB) in a representative healthy control. Points 1 to 5 represent anatomical landmarks (LMs) used for sub-segmentation analysis (see Material and Methods).

**Figure 2:**
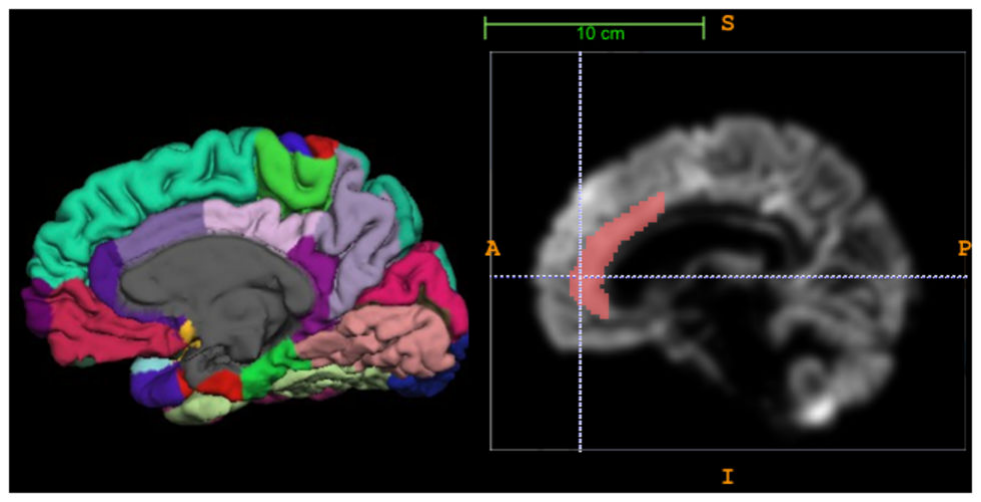
Delineation of the anterior cingulate cortex. a) Medial view of the right hemisphere in a healthy control with cortical parcellation according to Desikan Killiany, using Freesurfer software. Note the division of the cingulate cortex into anterior cingulate cortex (ACC) and posterior cingulate cortex, and the further division of the ACC into a rostral anterior division (dark purple) and the caudal anterior division (light purple). b) Illustration showing the anatomical mask used on VBM rendered images to delineate the ACC.

Navigation to manually trace ROIs for the DTT was done on FA maps in sagittal, coronal and transversal planes, using TrackVis. ROIs for the cingulum bundle were manually drawn using a “comprehensive” approach according to Catani and co-workers [25], consisting of one single ROI per hemisphere covering the entire cingulum bundle, including its ventral parts. This approach aims to include streamlines running along the cingulum bundle as well as shorter streamlines, thought to represent U-fibers, passing through the same anatomical region [25]. For all subjects, a NOT ROI was placed in the midline to exclude commissural streamlines which were not included in the present analysis. Also, in cases where apparent artifacts were generated, these were excluded by additional, manually drawn, NOT ROIs. All image analysis was done blinded for diagnosis. Inter and intrarater reliability for this tracking procedure was assessed on 10 cases (five patients and five controls) from the study cohort, using volume of tract in ml as the quantitative measure. In order to not limit the analysis to values of the whole tracts only, we used in-house developed software, Quantitative Tractography Evaluation (QuTE), which allows extraction of diffusion parameters in each cross section along a bundle of streamlines [32]. To allow for group comparisons, the extracted bundles of streamlines were normalized within each of the groups based on relevant anatomical locations, as shown in Figure 1. For the normalization in QuTE, anatomical landmarks (LMs) were used. The LMs were defined in TrackVis as single voxel ROIs, drawn in five positions along the graphical rendering of the cingulum bundle. The LMs were defined with the purpose of making a spatial normalization of all subjects, and to provide a basis for a relevant comparison between corresponding anatomical positions (see Figures 1, 2A and 2B). The LMs can be seen in Figure 1. LM1, LM2 and LM3 were positioned in the dorsal part of the cingulum bundle, while LM4 and LM5 were positioned in the ventral part of the cingulum bundle: LM1: in the transversal plane, in the most anterior point of the corpus callosum; LM2: in a coronal plane, the most dorsal point of the CC; LM3: in a coronal plane, the last slice where the corpus callosum can be seen posteriorly; LM 4: in the transversal plane, in the first slice where the corpus callosum can be seen in the dorsal/ventral axis; and LM5: in the coronal plane, in the slice in between where LM2 and LM 4 were placed. In cases where there was a choice of several voxels within the slice, the voxel with the highest intensity on FA map was chosen. The LMs were then used to normalize the bundles of streamlines that represent the cingulum bundle tracts. Anatomical segments between the LMs and diffusion parameters for each position along the bundles of streamlines could thereby be averaged within the groups. The analysis method was first employed for group comparisons between the patient group and the group of healthy controls, to examine whether results from the QuTE analysis were in concordance with previous studies. Second, mean values of DTI parameters of the segment between the landmarks relevant for the comparison with the ACC (LM1 and LM2) were extracted for each individual patient for further quantitative analysis.

### Cortical analysis

Analysis of cortical parameters was performed on 3D images. We used two different approaches to quantify cortical pathology. The first approach was to assess cortical thickness using Freesurfer software, version 5.1.0 (http://surfer.nmr.mgh.harvard.edu/). This included skull stripping, automated Talairach transformation, segmentation of the subcortical white/gray matter structures, intensity normalization, automated topology correction, and registration to a spherical atlas. This was followed by parcellation of the cerebral cortex into units based on gyral and sulcal structure [33,34]. The pipeline generated cortical thickness (mean, in mm, of each area) and cortical volumes (mm^3^ of each volume). All of the images were visually inspected after each step of processing. The anatomical parcellation according to Desikan Killiany map [33] divides the ACC into a rostral anterior division and a caudal anterior division (Figure 2A), and for the present study a mean between these two divisions was used, with the purpose of including the entire ACC. Results after parcellation were accepted if they included the ACC, and in the cases of a present paracingulate gyrus, the ACC only or ACC and the paracingulate. To partly avoid the problem that the parcellation scheme does not always discriminate between the cingulate and paracingulate cortex, we used only cortical thickness for further analysis.

The second approach was to generate measures of mean gray matter density of the anterior cingulate for each individual subject using FSL software (version 4.1) [35]. This approach, which we have used previously [36], is a modification of the standard FSL-VBM which allows for the comparison of individual data instead of group comparisons only. Brain extraction of T1 images was performed using BET [37], followed by tissue type segmentation with FAST 4.1 [38]. The segmented gray-matter images were then aligned to MNI52 standard space using the affine registration tool FLIRT [31,39]. The resulting images were averaged to create a study-specific group (thus an average image of all included subjects). Next, gray matter images of each individual subject were non-linearly registered to this study specific template. We subsequently applied an anterior cingulate mask, as previously described [36], to the gray matter data of each subject with AFNI software (http://afni.nimh.nih.gov/afni/) (Figure 2B). With this procedure, the mean gray matter density of the ACC for each subject was obtained.

### Statistical analysis

To test for possible differences in demographical variables, bvFTD and HC groups were compared with the Mann–Whitney U test (independent sample) for age, education, and the χ^2^ test for sex distribution, with the significance level set to 0.05. Q–Q plot analysis and the Shapiro-Wilk test on all MRI-derived variables showed that parametric analysis could be used for the data. Intra and interrater reliability for the manual tractography was assessed using the intra-class correlation coefficient (two way, absolute agreement, single measures). Cortical and white matter parameters in bvFTD and healthy controls were first compared using a two-tailed Student’s t-test, with a significance level of p=0.008 after Bonferroni correction for 6 different parameters (0.05/6). Effect size was calculated according to Cohen and expressed as Cohen’s *d*. Discriminatory power for each variable was examined using ROC analysis with an AUC calculation, here also with a significance level corrected for multiple comparisons to p=0.008. The results from the ROC analysis were validated using a binary logistic regression analysis, in a model (ENTER) with diagnosis/no diagnosis as outcome, the white matter diffusion parameters and cortical parameters, respectively, together with age, sex and education as covariates. Since this was a secondary analysis, a significance level of p=0.05 was retained despite multiple comparisons. This also applies to the statistical comparison between correct/incorrect classified cases, which was performed with McNemar test. Optimal cut off value for classification was derived by the Youden index. Correlation between volumetric and DTI parameters was analyzed using two-tailed Pearson correlation, again with a significance level of p=0.05. Statistical comparison of the WMH (ordinal data) was performed with the Mann-Whitney test. All analysis was performed with SPSS software version 19.0.0 (SPSS Inc., Chicago, IL) except calculation of optimal cut off value, for which MedCalc version 11.5.1 was used (MedCalc Software, MariaKerke, Belgium).

## Results

Demographic data for HC and bvFTD patients are shown in Table 1. There were no significant differences in age (p=0.475) or in sex distribution (p=0.79) between patients and controls, but there was a significant difference in education (p=0.009). Our patient cohort is similar to the previous DTI imaging studies on bvFTD regarding severity of disease (as measured with CDR, MMSE, duration), and sex distribution. Our cohort in slightly older, in accordance with several other studies on bvFTD [40]. Intra-class correlation coefficient of the intra-rater reliability (author AFS) was 0.947 (0.808-0.981), p<0.001, and of the inter-rater reliability 0.932 (0.760-0.982), p<0.001. There was no statistically significant difference in the amount of WMH between bvFTD and controls according to the Fazekas scale either for periventricular hyperintensities (PVH, p= 0.272) or white matter hyperintensities (DMWH, p=0.754), or the Wahlund scale (frontal lobes, p=0.248).

### Diffusion tensor imaging parameters

**Figure 3:**
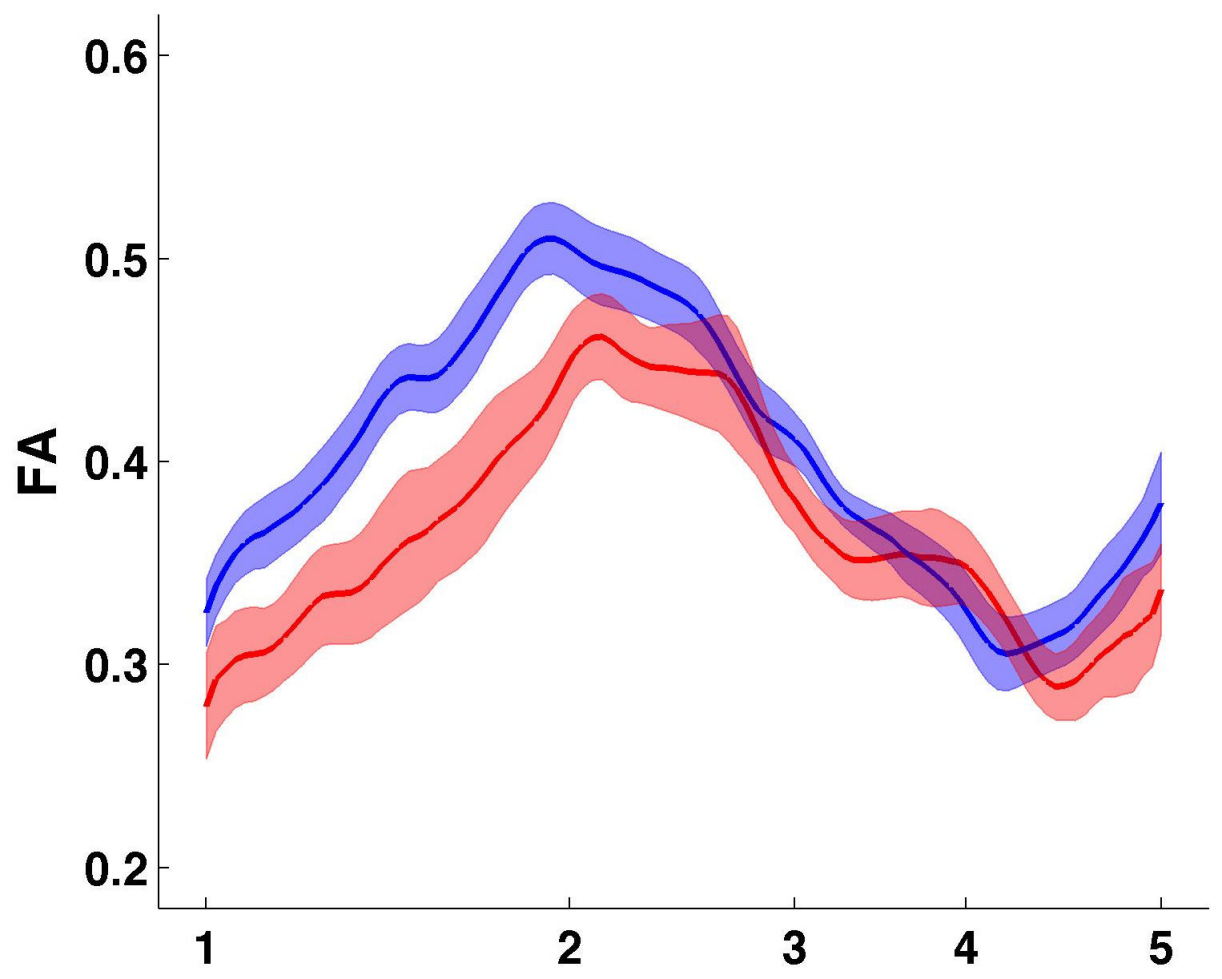
Quantitative Tractography Evaluation of the cingulum bundle. Graphical representation of the Quantitative Tractography Evaluation (QuTE) of the cingulum bundle. Y-axis: fractional anisotropy (FA) of the left and right cingulum bundle (mean). Red color: patients with behavioral variant frontotemporal dementia, blue color: healthy controls. The central line represents the mean, fields indicate +/- 1 standard error. X-axis refers to the numbers of the anatomical landmarks used to subsegment the cingulum bundle (see Figure 1 and Material and Methods).

The QuTE analysis of the cingulum bundle showed, as expected from previous studies, lower fractional anisotropy (FA) values in the anterior part of the cingulum bundle in patients with bvFTD compared to healthy controls, but not in the posterior or ventral part (Figure 3). This portion (between LM 1 and LM 2, denoted as LM12) was extracted for further analysis. In the healthy controls, in LM12 was higher in the left hemisphere compared with the right. In the numerical comparison between bvFTD and controls, the left hemisphere was slightly more affected then the right (Table 2) in all diffusion parameters. As expected, there was a lower FA and mean diffusivity (MD), and higher axial (aD) and radial diffusivity (rD) when comparing patients with bvFTD and healthy controls, with large effect sizes (Table 2). rD was the parameter that showed the greatest difference between bvFTD and healthy controls, followed by MD. Differences were statistically significant for all parameters except for aD LM12 of the right hemisphere.

**Table 2:** Parameters of cortical and white matter integrity in patients with behavioral variant frontotemporal dementia (bvFTD) and healthy controls (HC).

Parameter	HC	bv FTD	effect size	p-value	AUC
ACC thickness lh	2.76 (SD 0.21)	2.6 (SD 0.26)	0.57	0.085 NS	0.66 NS
ACC thickness rh	2.72 (SD 0.21)	2.66 (SD 0.31)	0.54	0.49 NS	0.59 NS
ACC VBM lh	0.54 (SD 0.041)	0.44 (SD 0.091)	1.67	<0.001*	0.78^#^
ACC VBM rh	0.55 (SD 0.042)	0.47 (SD 0.070)	1.42	0.001*	0.82^#^
FA LM12 lh	0.47 (SD 0.034)	0.39 (SD 0.053)	1.84	<0.001*	0.92^#^
FA LM12 rh	0.39 (SD 0.038)	0.33 (SD 0.047)	1.41	0.001*	0.81^#^
MD LM12 lh	0.81 (SD 0.036)	0.92 (SD 0.070)	2.1	<0.001*	0.94^#^
MD LM 12 rh	0.85 (SD 0.036)	0.96 (SD 0.12)	1.41	0.003*	0.87^#^
aD LM12 lh	1.24 (SD 0.055)	1.32 (SD 0.062)	1.37	0.002*	0.82^#^
aD LM12 rh	1.21 (SD 0.049)	1.32 (SD 0.11)	1.34	0.012 NS	0.86^#^
rD LM12 lh	0.59 (SD 0.041)	0.73 (SD 0.071)	2.45	<0.001*	0.97^#^
rD LM12 rh	0.67 (SD 0.047)	0.82 (SD 0.12)	1.78	0.002*	0.96^#^

Effect size: Cohens *d* P-values refers to Student’s t test, with *: significant at the p 0.008 level and NS: not significant. AUC: area under curve in ROC analysis to discriminate HC and bvFTD. ^#^: AUC significantly different from random (at p 0.008 level). ACC thickness: thickness of anterior cingulate cortex (ACC), in mm. ACC VBM: gray matter voxel integrity of ACC. FA: fractional anisotropy, MD: mean diffusivity, rD: radial diffusivity, aD axial diffusivity, all between landmark 1 and 2 (LM12) from the QuTE analysis. rh, lh: right and left hemisphere. Values are mean, +/- one standard deviation.

### Cortical parameters

Data are presented in Table 2. For the Freesurfer analysis, 6 hemispheres were excluded because of inaccurate parcellations. In the VBM FSL analysis, two controls were excluded because of technical problems. VBM showed statistically significant reductions in bvFTD compared with healthy controls, with large effect sizes, with the left hemisphere showing greater involvement. In the case of the thickness, differences were only moderate and statistically non-significant. However, despite no macroscopic frontal and/or temporal atrophy on MRI being determined by neuroradiologist, the four cases of possible bvFTD displayed a slightly significant cortical thinning compared with controls (p=0.022 on Student’s t-test).

### Discriminatory potential of DTI versus cortical integrity parameters

All AUC are significantly different from random (0.5) except thickness in both hemispheres. The cortical parameter with best discriminatory potential between bvFTD patient and controls was VBM, with an AUC of 0.78 for the left hemisphere and 0.82 for the right hemisphere. AUC was larger for several diffusion parameters. In the left hemisphere: FA (AUC=0.92), MD (AUC=0.94), rD (AUC=0.97), and in the right MD (AUC=0.87), rD (AUC=0.96) and aD (AUC=0.86). Differences between AUC of VBM and diffusion parameters were generally larger in the left hemisphere than in the right, and further analysis is therefore based on the left hemisphere. In the binary logistic regression, all models predicted patient/non-patient status better than baseline expectance (56%) and the respective imaging parameter contributed significantly to the models, except for thickness, aD and rD. Thickness could correctly classify 78% of cases (p=0.117, B=3.54), VBM 83% of cases (p=0.049, B=51.0), FA LM12 84% (p=0.025, B=44.3), MD LM12 90% (p=0.017, B=-50.6), aD 88% (p=0.065, B =-22.4), and rD 92% (p=0.302, B =-114.1). Using other models for the binary logistic regression (Backward/Forward LR) did not change these results in any significant manner. The differences between the parameters when comparing correct classification/incorrect classification were statistically significant for MD vs. VBM (p=0.031), and borderline significant for FA vs. VBM (p=0.062). Statistical difference in classification potential was not assessed for aD and rD since these parameters did not survive in the binary logistic models. Based on the Youden index optimal sensitivity/specificity was 100/77% for MD and 92/81% for FA. Post-hoc analysis of the patients classified as “possible” and “probable” bvFTD separately did not change the relationship between parameters in a significant manner, with possible cases having an AUC for MD of 0.86 and thickness 0.57, probable patients having AUC for MD of 0.94, thickness 0.74.

### Correlations between DTI and cortical integrity parameters

In the left hemisphere, VBM was moderately correlated with FA (r=0.516, p=0.002) and MD (r=-0.668 p<0.001), rD (r=-0.696, p<0.001),) and aD (r=-0.610, p=0.001). VBM and cortical thickness showed a large and significant correlation in the right hemisphere (r=0.527, p=0.002), but moderate correlation (r=0.332) and only with borderline significance (p= 0.068) for the left hemisphere.

## Discussion

In this study we compared the ability to discriminate between patients with bvFTD and healthy controls, of MRI parameters reflecting white and gray matter pathology of the anterior cingulate cortex and cingulum bundle respectively. Our results show that DTI parameters are on the whole more discriminative that those of cortical integrity; however, the differences are modest, with the best parameter of cortical pathology having an AUC of 0.83 and the best DTI parameter 0.92 to 0.95. Could these differences be clinically relevant? The international criteria for the diagnosis of bvFTD require pathological neuroimaging for a diagnosis of probable bvFTD [2], and, given that structural MRI is part of the standard diagnostic work up, we believe that such a modest gain could be of clinical benefit.

A number of studies have shown affected DTI parameters in bvFTD that roughly correspond to regions of cortical volume loss, but only one has compared DTI parameters and parameters of cortical integrity in the same individual cases [17]. The differences of our study are that we a) use an *a priori* chosen anatomical region b) compare the corresponding cortical and white matter integrity of this chosen region, c) analyze it with more precise anatomical localization, using deterministic diffusion tensor tractography, and c) use two different methods to assess cortical pathology. We have chosen the ACC/cingulum bundle, since this is one of the earliest regions affected in bvFTD and the region most consistently affected in bvFTD [18–20] making it of critical importance in diagnostics, particularly in early disease stages, and because it has a close correspondence between the cortex and the white matter tract. Our results are in accordance with the study of Zhang [17], which showed increased discriminatory potential in AUC/logistic regression analysis of DTI compared with VBM for the frontomedial area, but there are also some differences. In both studies, rD appears to be the most sensitive DTI parameter but in our study, rD did not contribute significantly to the binary logistic regression model which included demographical covariates. In several other studies of bvFTD, rD is the diffusion parameter that is most sensitive to change, more than FA and MD, while less change is seen in aD [6,7]. However, the validity of rD has been questioned [41] and it is possible that this parameter is more sensitive for partial volume effects. A second study has assessed the discriminatory potential of DTI in bvFTD versus controls [9], using random forest analysis of tract-based spatial statistics FA data, but not compared directly with cortical integrity. Despite showing significant changes of DTI parameters in the cingulum bundle, the cingulum bundle was not one of the most discriminative tracts, in contrast to the anterior corpus callosum and the uncinate fasciculus.

There are other ways to measure cortical atrophy in addition to those we have employed. We chose these two methods since, while VBM and cortical thickness generally show a good correlation, this is not always the case, particularly for the ACC [36]. In our study, the correlations between VBM of the ACC and thickness was different for the right (r=0.527, p=0.002) and the left (r=0.332, p=0.068) hemisphere. We interpret this finding as due to differences in cingulate/paracingulate gyrification and the way that the two methods handle them. In the right hemisphere, where a paracingulate is uncommon, the same structure is measured by both methods, resulting in better correlation. In the left hemisphere, since the parcellation scheme employed by our Freesurfer analysis does not always distinguish between cingulate and paracingulate [33], the methods will have a lower correlation. In both hemispheres, the VBM method was clearly more sensitive for pathology and showed a better discriminatory potential than measurements of cortical thickness. The results of the FreeSurfer parcellation were not always accurate, and 6 hemispheres had to be excluded. One case (2 hemispheres) was a bvFTD patient with moderate-severe atrophy, which could have influenced the results for ACC thickness. Retrospective studies have shown considerably lower sensitivity and specificity for morphological MRI than our results of an AUC of 0.83 imply, but this is probably due to the fact that quantitative volumetric measurements are more sensitive than semi-quantitative standard radiological examination.

There are also various ways of analyzing tractographic data. When only looking at mean values for the entire reconstructed tract, there is a risk of being too insensitive to changes in various sub-regions of the tract. Thus, different researchers have used different ways to conduct a cingulum bundle tractography in the literature, often with some subsegmentation [6,42] into anterior, dorsal and ventral parts. However, it is not entirely clear what these tractographical subsegmentations correspond to morphologically, and they do not include shorter streamlines. Also there is the option of assumption-free, non-tractographical DTI methods such as voxel-wise comparisons on standardized brain templates, using VBM and Tract-Based Spatial Statistics, which are not bound by anatomical boundaries and have the possibility of detecting change in sub-regions of the cingulum bundle [8,9]. We believe that our QuTE method can combine some advantages of the two approaches, using tractographic stringency in delineating the neuroanatomy with the possibility to detect changes with sub-region analysis [32]. A possible source of bias in this study is partial volume effects, due to contributions from neighboring structures other than the cingulum bundle to the information in each voxel [43]. Partial volume effects are a particular problem for tracts bordering the CSF, which is not the case for the cingulum bundle, but in our study partial volume effects could arise because of the cingulum bundle’s two adjacent structures, the ACC and the corpus callosum, which have other diffusion properties [44]. As can be seen in Table 2, there is an asymmetry of the DTI parameters (mainly FA and rD) among the healthy controls with left hemisphere FA > right hemisphere FA. This asymmetry, which is of the same magnitude as the difference between bvFTD and healthy controls in the same hemisphere, is previously described in detail, although unexplained [42].

How could methods for detecting white matter changes be more sensitive than measures of cortical integrity? Cortical volume loss in neurodegenerative disease probably represents loss of single neurons (soma) and neuropil (corresponding dendrites, axons). This volume loss, however, is partially compensated by processes such as the gliotic response and vacuolisation. The eigenvalues in DTI and secondary constructs seem to mirror more subtle changes that will not initially affect gross volume [45]. In frontotemporal dementia, white matter changes are characteristically those of astrocytic gliosis and myelin loss, without cell loss, a pathology also affecting the U-fibers [14–16]. The characteristic neuropathological protein depositions (tau, TDP-43) extend into the white matter underlying the affected cortex [46,47]. Thus, the neuropathology that DTI parameters represent most likely are either demyelination and/or astrocyte/microglia activation, which was shown in a post-mortem DTI study of FTD [16], or a possible direct effect of the neuropathological protein depositions. Vascular burden in the white matter could however be a potential confounder in our study, as in other studies of DTI in neurodegenerative disease. As in previous studies [12,17], we found a correlation between DTI parameters and volumetric parameters, indicating that these are measures of the same pathologic process, i.e. neurodegeneration. It is probable that the relative change in parameters reflecting cortical integrity and diffusion not only varies depending on the methods employed, but also on the particular morphological process, which anatomical region is being studied, and possibly the underlying molecular pathology of the particular disease. The lack of definitive diagnosis (through postmortem neuropathological examination or the presence of a known autosomal dominant mutation) is a limitation of our study.

## Conclusion

In conclusion, we show that the assessment of white matter integrity of the cingulum bundle with diffusion tensor tractography is a powerful tool for separating individual patients with bvFTD and controls. DTI parameters are better than measures of cortical atrophy, the differences being modest but potentially clinically useful. Our results provide an impetus to explore this subject further, in bvFTD and other neurodegenerative diseases.
